# Vitrification preserves chromatin integrity, bioenergy potential and oxidative parameters in mouse embryos

**DOI:** 10.1186/1477-7827-11-27

**Published:** 2013-04-03

**Authors:** Nicola A Martino, Maria E Dell’Aquila, Rosa A Cardone, Bence Somoskoi, Giovanni M Lacalandra, Sandor Cseh

**Affiliations:** 1 Veterinary Clinics and Animal Productions Unit, Department of Emergency and Organ Trasplantation (DETO), University of Bari Aldo Moro, Valenzano, Bari, Italy; 2 Department of Bioscience, Biotechnology and Biopharmaceutics, University of Bari, Aldo Moro, Bari, Italy; 3 Department and Clinic of Obstetrics and Reproduction, Szent Istvan University, Budapest, Hungary

## Abstract

**Background:**

The aim of this study was to evaluate the effects of vitrification on morpho-functional parameters (blastomere/chromatin integrity and bioenergy/oxidative potential) of mouse preimplantation embryos.

**Methods:**

In vivo produced mouse (4/16-cell, morulae and blastocyst-stage) embryos were randomly divided into vitrification and control groups. For vitrification, embryos were exposed to a 2-step loading of ethylene glycol and propylene glycol, before being placed in a small nylon loop and submerged into liquid nitrogen. After warming, the cryoprotectants were diluted by a 3-step procedure. Embryo morphology, chromatin integrity and energy/oxidative status were compared between groups.

**Results:**

Vitrification induced low grade blastomere cytofragmentation (P < 0.05) and low chromatin damage only in embryos at the morula stage (P < 0.001). Mitochondrial (mt) distribution pattern was affected by vitrification only in early embryos (P < 0.001). Mitochondrial activity did not change upon vitrification in morula-stage embryos but it was reduced in blastocyst-stage embryos (P < 0.05). Intracellular ROS levels significantly increased in embryos at the morula and blastocyst stages (P < 0.001). Colocalization of active mitochondria and ROS increased only in vitrified blastocysts.

**Conclusions:**

In conclusion, this study elucidates the developmentally-related and mild effects of vitrification on morphology, nuclear and bioenergy/oxidative parameters of mouse embryos and demonstrates that vitrification is a suitable method for preserving predictive parameters of embryo ability to induce a full-term pregnancy.

## Background

Vitrification is an alternative approach to freezing, which avoids the formation of ice crystals in the intracellular and extracellular space [1-3]. Vitrification is the solidification of a solution at low temperature, a process achieved by a combination of a high concentration of cryoprotectants (CPAs,4-8 M) and an extremely high cooling rate [4,5].

Appropriate mitochondrial (mt) distribution and membrane potential in embryos are very important for developmental potential and for a variety of cellular activities, including ATP synthesis and specific cell functions [6,7]. However, there is little information available about the effects of vitrification on chromatin integrity, mt dynamics/distribution and ROS production in oocytes/embryos. Zhao et al. 2009 [8] reported that, in mouse two-pronuclear stage embryos, vitrification reduces the rate of mt ring formation around pronuclei, an event which may affect the syngamy of male and female pronuclei and subsequent embryo development. Shi et al. [9] reported that vitrification alters the mt distribution pattern in porcine MII oocytes.

It has been reported that oxidative stress (OS) may be an important mechanism underlying the toxic effects of cryopreservation procedures, which may then trigger the apoptosis cascade leading to a decrease in the survival and developmental rate of gametes and embryos after thawing [10-21]. Oxidative stress occurs if a disequilibrium takes place between reactive oxygen species (ROS) production and antioxidative capacity of the cell [14] and it has also been implicated in the etiology of some forms of female infertility [15]. Mitochondria represent the major source of ROS and they are produced in a stepwise process with a final reduction of O_2_ to H_2_O during oxidative phosphorylation, in particular at the level of complex I and III [16]. Under physiological conditions, ROS are neutralized by an elaborate defence system consisting of enzymes such as catalase, superoxide dismutase, glutathione peroxidase or reductase and numerous non enzymatic antioxidants such as vitamin C, E, A, pyruvate, glutathione, ubiquinone, taurine and hypotaurine [17]. Thus, any perturbation in mt activity or in the activity of scavenger systems can lead to profound alterations in ROS production, OS induction, and mt cytochrome c release, which is an important step for apoptosis [18]. Embryos, as other aerobic cells, produce ATP and ROS by means of mt oxidative phosphorylation.

To our knowledge, the effects of vitrification on embryo morphology, chromatin integrity, mt distribution, energy status and ROS production in different developmental stages have not been documented to date. Therefore, the aim of the present study was to investigate the effects of vitrification on morpho-functional parameters of mouse embryos at different developmental stages. Blastomere morphology, expressed in terms of blastomere cytofragmentation; chromatin integrity, assessed as the rate of embryos showing low or high grade chromatin damage; mt distribution pattern, if either heterogeneous or homogeneous, indicating strong or weak energy condition, respectively; mt activity and intracellular levels of ROS, expressed as fluorescent intensities of specific probes, and mt/ROS colocalization, were examined. Our study could contribute to understanding whether, during embryo development, mt display dynamic tubular networks undergoing fission, fusion, organization in granules and tubules and intracellular movements as it has been demonstrated within the oocyte and whether any change in these events upon the use of cryopreservation procedure.

## Methods

### Embryo recovery and in vitro culture

Procedures with animals were performed following good veterinary practice for animal welfare according to Hungarian national laws in force. The protocol of the animal experiment was approved by the Ethical Committee of the Faculty of Veterinary Science Szent Istvan University of Budapest. Embryos were produced as reported by Klambauer et al., 2009 [19]. Briefly, eight weeks old CB6F1 female mice (Institute of Oncology, Animal Care Facility, Budapest, Hungary) were superovulated by 10 IU eCG i.p., followed by 10 IU hCG i.p. 48 h later, in order to induce the final maturation of the oocytes and ovulation. After the hCG injection the females were paired with males (1 female/male), then 20 to 24 h later the embryos were collected (Day 1). Until vitrification or analysis (fresh control embryos), embryos were cultured in G1 medium (Vitrolife, Goteborg, Sweden) at 37.5°C with 6.5% CO_2_ and maximal humidity in air for further 20 to 96 h. Only morphologically normal embryos, being in cleavage stage (4- to 16-cell) or at the morula or blastocyst stage, were randomly destined to either vitrification or fresh control groups.

### Vitrification

Embryo vitrification was performed with the VitroLoop vitrification procedure as previously described [19] and unless otherwise specified, all materials were provided by Vitrolife. Briefly, embryos were exposed to a 2-step loading of the CPA solution, ethylene glycol (EG) and propylene glycol (PG), before being placed on a thin filmy layer formed from the vitrification solution in a small nylon loop, then they were rapidly submerged in liquid nitrogen (LN_2_). Vitrification was carried out in RapidVit Cleave vitrification solutions (Vitrolife; solution 1: holding medium, solution 2: equilibration medium, and solution 3: vitrification medium) and embryos were manipulated in 4-well culture dish (Nunc Intermed, Roskilde, Denmark) held on a warming plate at 37°C. The holding or basic solution is based on G-MOPS™ and was supplemented with gentamycin and human serum albumin (HSA, 5 μg/ml). Both the equilibration and the vitrification media are based on the holding/basic solution, but the equilibration medium was supplemented with 8% EG + 8% PG and the vitrification solution was enriched with 16% EG, 16% PG, F (Ficoll, F-400, 10 mg/ml) and S (Sucrose, 0.65 mol/l). All manipulations of the embryos during their preparation for vitrification were carried out at 37°C (on a heated stage). Embryos were suspended from solution 1 into the equilibration medium (solution 2) for 2 min. Thereafter, they were transferred and washed quickly in small drops of vitrification medium (solution 3). The cryoloop was dipped into the vitrification medium to create a thin filmy layer of the solution on the nylon loop where embryos (max 3 embryos) were quickly transferred from the vitrification medium. Within 30 sec of suspension in the vitrification medium, the loop with the embryos was plunged into LN_2_. Embryos were warmed and rehydrated by a 3-step dilution of the CPA performed at 37°C. At warming, the embryos were moved through a series of G-MOPS™ solutions containing the S in decreasing concentrations (warming solution 1: 0.65 mol/l; 30 sec, warming solution 2: 0.25 mol/l; 1 min, warming solution 3: 0.125 mol/l; 2 min and warming solution 4: 0.0 mol/l; 5 min) (RapidWarm Cleave; Vitrolife, Goteborg, Sweden).

### Mitochondria and ROS staining

Fresh and vitrified-warmed embryos underwent mt and ROS staining following the procedure by Ambruosi et al., 2011 and Martino et al., 2012 [20,21]. Embryos were washed three times in PBS with 3% bovine serum albumin (BSA) and incubated for 30 min in the same medium containing 280 nM MitoTracker Orange CMTM Ros (Molecular Probes M-7510, Oregon, USA) at 38.5°C under 5% CO_2_ in air. The cell-permeant probe contains a thiol-reactive chloromethyl moiety. Once the MitoTracker probe accumulates in the mitochondria, it can react with accessible thiol groups on peptides and proteins to form an aldehyde-fixable conjugate. This cell-permeant probe is readily sequestered only by actively respiring organelles depending on their oxidative activity [22,23]. After incubation with MitoTracker Orange CMTM Ros, embryos were washed three times in PBS with 0.3% BSA and incubated for 15 min in the same media containing 10 mM 2’,7’-dichlorodihydrofluorescein diacetate (DCDHF DA). The non-ionized DCDHF DA is membrane permeant and therefore is able to diffuse readily into cells. Once within the cell, the acetate groups are hydrolysed by intracellular esterase activity forming 2’,7’-dichlorodihydrofluorescein (DCDHF) which is polar and thus trapped within the cell. DCHF fluoresces when it is oxidized by H2O2 or lipid peroxides to yield 2’,7’-dichlorofluorescein (DCF). The level of DCF produced within the cells is linearly related to that of peroxides present and thus its fluorescent emission provides a measure of the peroxide levels [24]. After incubation, embryos were washed three times in prewarmed PBS without BSA and fixed with 3.7% paraformaldehyde solution in PBS. All procedures after thawing/warming were performed within 1 hour. Embryos were kept in fixative at 4°C for no longer than two to three days. The organelle-specificity of the mt probe was assessed, as reported by Valentini et al., 2010; [25], in control samples which were imaged after incubation in MitoTracker Orange and further incubation for 5 min in the presence of 5 mM of the mt membrane potential (Delta Psi)-collapsing uncoupler carbonyl cyanide 3-chloro phenylhydrazone (CCCP; Molecular Probes), which inhibits mt respiratory activity thus reducing fluorescence intensity. Particular attention was paid to avoid sample exposure to the light during staining and fixing procedures in order to reduce photobleaching.

### Embryo morphology assessment

Embryos at the different examined developmental stages were recovered from vials and their morphological appearance was assessed by evaluating blastomere cytofragmentation following the criteria described in previous studies in the mouse as well as in other species [26,27]. Embryos were examined at 400x magnification phase contrast microscopy and scored as either unfragmented (Grade 0), or with fragmentation graded into three categories as reported by Van Soom et al., 2003 and Han et al., 2005 [26,27]. Grade 1 fragmentation was defined as the presence of one or more cytofragments smaller than the size of a polar body and often clustered at one or both poles or in the crevice between blastomeres. Grade 2 fragmentation was defined as the presence of many more fragments, with total volume of fragments comprising an equivalent of less than one-half the volume of a blastomere. Grade 3 fragmentation was assigned to embryos with many large fragments, with the total volume of fragments being approximately one-half of one blastomere or greater.

### Detection of the nuclear chromatin status

To evaluate nuclear chromatin, embryos were stained with 2.5 μg/ml Hoechst 33258 in 3:1 (v/v) glycerol/PBS, mounted on microscope slides, covered with cover-up micro slides, sealed with nail polish and kept at 4°C in the dark until observation [20]. Nuclear chromatin status was observed under a Nikon Eclipse 600 fluorescent microscope equipped with B2A (346 nm excitation/ 460 nm emission) filter. Embryos were classified as normal (grade A) when the presence of a regular-shaped nucleus inside each blastomere was observed. The formation of micronuclei and lobulated nuclei was considered as signs of chromatin damage [28]. Embryos showing 0 to 20% affected blastomeres were classified as grade B and embryos with more than 20% affected blastomeres were classified as grade C.

### Mitochondrial distribution pattern and intracellular ROS localization

For mt distribution pattern evaluation, embryos were observed at 600 × magnification in oil immersion with a Nikon C1/TE2000-U laser scanning confocal microscope. A helium/neon laser ray at 543 nm and the G-2 A filter (551 nm exposure/576 nm emission) were used to observe the MitoTracker Orange CMTM Ros. An argon ions laser ray at 488 nm and the B-2 A filter (495 nm exposure/519 nm emission) were used to observe the DCF. Scanning was conducted with 25 optical series from the top to the bottom of the embryo with a step size of 0.45 μm to allow three-dimensional distribution analysis. General criteria for mt pattern definition were adopted on the basis of previous studies in mouse and human oocytes and embryos [7,8,29,30], as well as in oocytes of other species [31]. Thus, an homogeneous/even distribution of small mt aggregates throughout the cytoplasm was considered as an indication of low energy cytoplasmic condition. Heterogeneous/uneven distribution of small and/or large mt aggregates indicated metabolically active cytoplasm. In particular, the accumulation of active mt in the peripheral cytoplasm (pericortical mt pattern) and/or around the nucleus (perinuclear and perinuclear/pericortical mt pattern, P/P) was considered as characteristic of healthy cytoplasmic condition. Embryos showing irregular distribution of large mt clusters unrelated to the specific cell compartments were classified as abnormal. To our knowledge, few studies have reported to date on intracellular ROS localization and levels in mouse embryos [32,33] and no studies on vitrified mouse embryos have been reported.

### Quantification of Mitotracker Orange CMTM Ros and DCF fluorescence intensity

Measurements of fluorescence intensities were performed in embryos having either heterogeneous (perinuclear/pericortical) or homogeneous (small aggregates) mt distribution pattern. Embryos showing abnormal mt distribution pattern were excluded from this analysis. In each individual embryo, the fluorescence intensity was measured at the equatorial plane, with the aid of the EZ-C1 Gold Version 3.70 software platform for Nikon C1 confocal microscope. A circle of an area (arbitrary value = 100 in diameter) was drawn in order to measure only the cytoplasmic area. Fluorescence intensity encountered within the programmed scan area was recorded and plotted against the conventional pixel unit scale (0–255). Quantification analysis was performed only on embryos at the morula or blastocyst stage. In fact, due to their round shape, late stage embryos allow the software set-up for quantification analysis as reported for oocytes [21]. Parameters related to fluorescence intensity were maintained at constant values for all evaluations. In detail, images were taken under fixed scanning conditions with respect to laser energy, signal detection (gain) and pinhole size.

### Mitochondria/ROS colocalization analysis

Colocalization analysis of mt and ROS was performed by using the EZ-C1 Gold Version 3.70 software. For each channel, the same threshold, set to the zero value, was used for the data set. Degree of colocalization was reported as a Pearson’s correlation coefficient quantifying the overlap degree between MitoTracker and DCDHF DA fluorescence signals [21,34].

### Developmental ability of vitrified embryos

In order to demonstrate whether vitrified embryos retain the developmental ability, a group of vitrified embryos were warmed and cultured in vitro as described above. Their developmental ability was compared with that of non vitrified control embryos. Moreover, the possible toxic effect of the cryoprotectant mixture was assessed, by comparing the developmental rate of non vitrified embryos exposed to cryoprotectants (non-vitrified/exposed) with that of non vitrified/non exposed embryos.

### Statistical analysis

For each examined embryo developmental stage, embryo morphology (the rates of embryos showing cytoplasmic fragmentation and chromatin damage) and the rates of embryos showing the different mt distribution patterns and ROS intracellular localization were compared between the fresh control and vitrified-warmed embryos by Chi square-analysis with the Yates correction for continuity. Fisher’s exact test was used when a value of less than 5 was expected in any cell. Mean values (mean ± SD) of mt and ROS fluorescence intensities, expressed as arbitrary density units (ADU) and mt/ROS colocalization, expressed as Pearson’s correlation coefficient, were compared by the Student’s t-test. Differences with P < 0.05 were considered statistically significant.

## Results

Two hundred and sixty-seven mouse embryos, 99 of which at the early stages of development (including 4/8-cell, n = 42 and 8/16-cell, n = 57), 122 at the morula stage (including 16/32-cell, n = 76 and >32 cell, n = 46) and 46 at the blastocyst stage, were randomly divided into vitrified (n = 118 embryos) and control (n = 149 embryos) groups.

### Vitrification increases low grade embryo blastomere cytofragmentation

The overall proportion of fragmented embryos (grade 1 + grade 2) was increased by vitrification (26% vs 5%, for vitrified-warmed vs control embryos, respectively; P < 0.05). However, most of fragmented embryos were found to be of low grade, i.e. of grade 1 (24% and 5% grade 1 fragmented embryos in vitrified and control groups, respectively; P < 0.05) and very few embryos were found to be of intermediate grade, i.e. of grade 2 (2% and 0% grade 2 fragmented embryos in vitrified and control groups, respectively; NS). No high grade fragmentation (grade 3) was found in either group.

### Vitrification increases low level embryo chromatin damage

**Figure 1:**
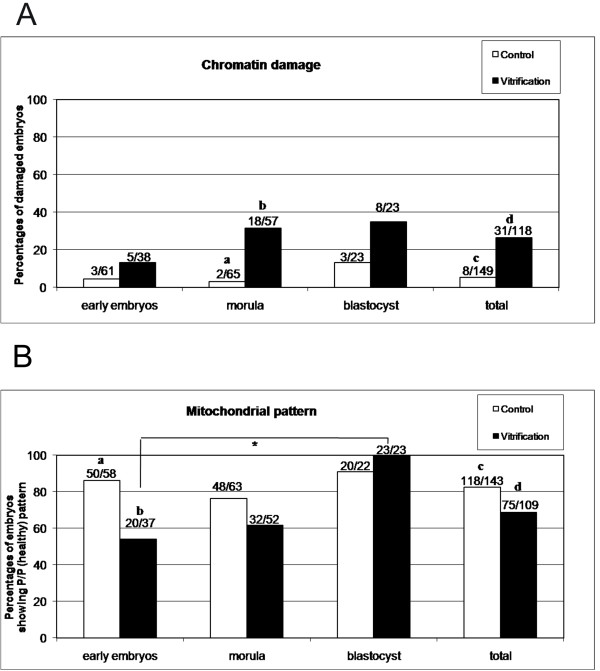
**Effects of vitrification on chromatin integrity and mitochondrial distribution pattern of mouse 4/16-cell, morula and blastocyst stage embryos.** Panel **A**: overall percentages of embryos (grade B + grade C) with damaged chromatin observed after vitrification (black bars), grouped according to their developmental stage, and compared with controls (white bars). In detail, in embryos at the morula stage, vitrification increased the rate of embryos showing chromatin damage whereas it had no effect in early embryos and in blastocysts. Chi square test: within each stage: a,b P<0.001; c,d P<0.05. Panel **B**:Percentages of embryos showing the P/P mt pattern observed after vitrification (black bars), grouped according to examined developmental stages, and compared with controls (white bars). In detail, in embryos at the 4/16-cell stage, vitrification reduced the rate of embryos showing P/P mt pattern whereas it had no effect in embryos at the morula and blastocyst stages. Chi square test: a,b P<0.001; c,d P<0.05; * P<0.001. Numbers of analyzed oocytes per group are indicated on the top of each histogram.

Vitrification induced low level nuclear chromatin damage (26% vs 5% in vitrified and control embryos, respectively; P < 0.05; Figure 1, panel A). Chromatin damage increased with embryo development and became statistically significant in embryos at the morula stage (P < 0.01). On the contrary, chromatin integrity was preserved in embryos at the blastocyst stage. Figure 1 (panel A) shows the overall (grade B + grade C) proportion of embryos with damaged chromatin observed after vitrification (black bars), grouped according to their developmental stage, and compared with controls (white bars). In Additional file 1: Figure S1, the proportions of embryos showing grade B (0 to 20%; panel A) or grade C (>20%; panel B) chromatin damage are detailed. As depicted in panel A, the proportions of grade B affected embryos were significantly higher after vitrification (24%) compared with controls (5%, P < 0.05) while vitrification had no effect on the rates of embryos showing grade C chromatin damage (3/118; 2.5%). In Figure 1 panel A and in the Additional file 1, values plotted on the y axis are the proportion of embryos with chromatin damage, thus complementary percentages are referred to normal (grade A) embryos.

### Vitrification preserves mt distribution pattern and ROS localization in mouse embryos

In Figure 1 (panel B), the percentages of embryos showing the P/P (healthy) mt pattern observed after vitrification (black bars), grouped according to their developmental stage, and compared with controls (white bars), are reported. Apparently, vitrification reduced the ratio of embryos showing P/P mt pattern compared to controls (overall data: P < 0.05). However, the developmentally-related data showed that vitrification significantly reduced the ratio of embryos showing the P/P mt pattern only at early stages (P < 0.001).

**Figure 2:**
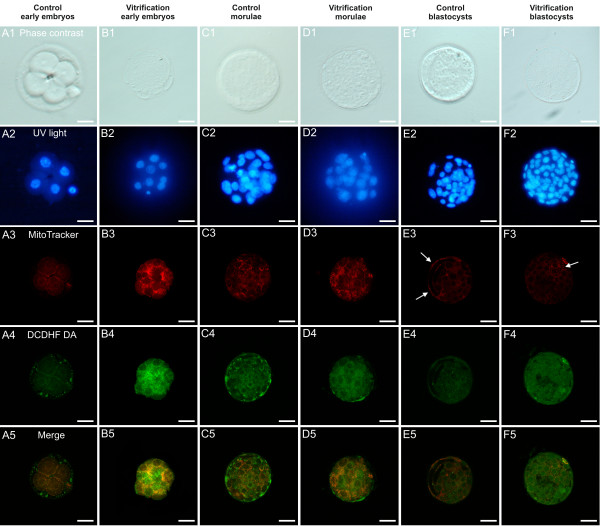
**Photomicrographs of fresh and vitrified/warmed mouse embryos at early (4/16-cell), morula and blastocyst stages of development as assessed for their nuclear chromatin and bioenergy/oxidative potential.** For each embryo, corresponding bright-field (phase contrast; lane 1), UV light (lane 2) and confocal images showing mt distribution pattern (lane 3), ROS localization (lane 4) and mt/ROS merge (lane 5), are shown. Representative photomicrographs of control non-vitrified and vitrified/warmed embryos at the 4- (row A) and 8-cell (row B), morula (rows C and D) and blastocyst stages (rows E and F), are shown. Nuclear chromatin was stained with Hoechst 33258.MitoTracker Orange CMTM Rosand DCDH FDA were used to label mitochondria and ROS, respectively. In control fresh early embryos and in morulae, in all blastomeres, there were detectable MitoTracker signals in the form of continuous rings around the nuclei and clusters of mitochondria at the cortex, namely perinuclear/pericorticalmt pattern (heterogeneous, healthy P/P mt pattern) (A3, C3). Vitrified early embryos(B3) showed a uniform/diffused (homogeneous) mt distribution pattern throughout the blastomere cytoplasm. In embryos at the morula and blastocyst stage, the mt pattern was apparently not affected by vitrification (D3 vs C3 and F3 vs E3). A higher number of red fluorescent spots is evident on the trophoectoderma (white arrows) compared with the inner cell mass, indicating differences in mt number/cell between these two embryo lineages and higher mt/number and aggregate formation in the trophoectoderma compared with ICM. This feature can be observed in embryos of both groups, thus it was not influenced by vitrification. ROS intracellular localization (lane 4) corresponded to the distribution pattern of mitochondria. In fresh control embryos, apart areas/sites of mt/ROS overlapping (merge, lane 5), intracellular ROS appeared diffused throughout the cytoplasm (A4, C4, E4). In vitrified (B4, D4 and F4) embryos, diffused MitoTracker and DCDH FDA labelling were evident throughout the cytoplasm. Scale bar represents 20 μm.

In Figure 2, representative photomicrographs of embryo blastomere cytoplasmic shape and texture, as observed after staining and fixing procedures (lane 1) and nuclear chromatin (lane 2) of vitrified-warmed embryos at different developmental stages (rows B, D and F) and their non-vitrified control counterparts (rows A, C and E), are shown. In addition, Figure 2 shows representative photomicrographs of heterogeneous and homogeneous mt distribution pattern (lane 3) with corresponding intracellular ROS localization (lane 4) and mt/ROS merge (lane 5) in vitrified-warmed and control embryos at the 4/16-cell, morula and blastocyst stages. In control fresh embryos (Figure 2, rows A, C, E), MitoTracker signals were detected in all blastomeres in the form of continuous rings around the nuclei and clusters of mitochondria at the cortex (heterogeneous, perinuclear/pericortical, P/P mt pattern), which has been reported in previous studies as an indication of healthy embryos (Zhao et al., 2009 for 2 pronuclear mouse embryos; [8]). Intracellular ROS appeared diffused throughout the cytoplasm in embryonic blastomeres at any stage of development and in both groups (vitrification and controls) apart areas/sites of mt/ROS overlapping (Figure 2, lane 4). In Figure 2, in embryos at the blastocyst stage, a higher number of red fluorescent spots was found on the trophoectoderm compared with the inner cell mass (ICM; E3 and F3), indicating differences in mt number or activation status per cell, between these two embryo lineages. Possibly, an higher mt number and/or aggregate formation of active mitochondria is found in the trophoectoderm compared with ICM. This feature was observed in all groups (23% and 22% for control and vitrified embryos: NS) and thus was not influenced by vitrification procedure.

### Vitrification alters mt activity and ROS levels but preserves mt/ROS colocalization in mouse preimplantation embryos

**Figure 3:**
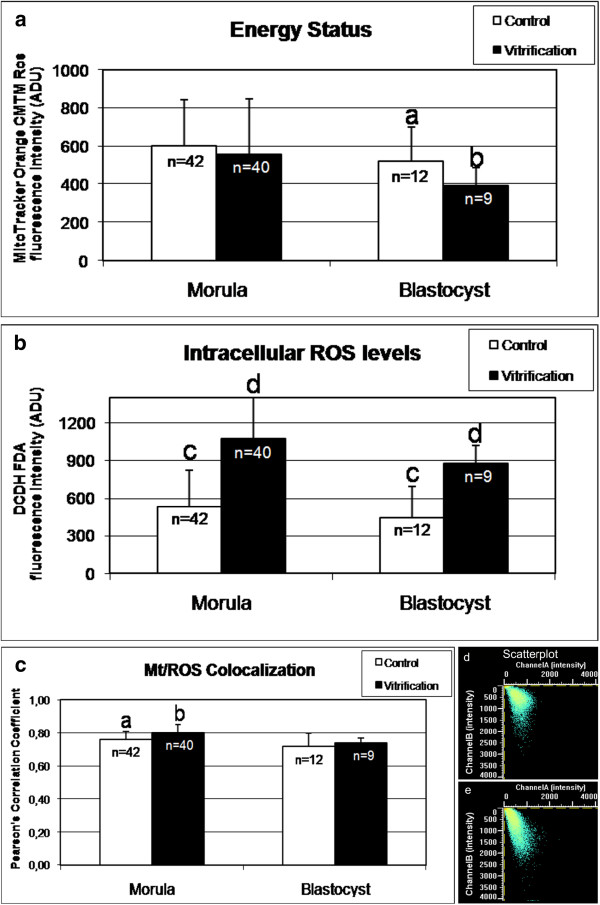
**Effects of vitrification on mitochondrial activity, intracellular ROS levels and mt/ROS colocalization in single mouse morulae and blastocysts.** In each group, energy status and ROS intracellular levels are expressed as mean±SD of Mitotracker Orange CMTM Ros (panel **a**) and DCF (panel **b**) fluorescence intensity of individual embryos in arbitrary densitometric units (ADU). In each group, mt/ROS colocalization is expressed as mean±SD of Pearson’s correlation coefficient of individual embryos (panel **c**). Representative mt/ROS colocalization scatterplots graph of a fresh (panel **d**) and a vitrified (panel **e**) blastocyst are shown. In embryos at the morula stage, mt activity did not change after vitrification whereas in embryos at the blastocyst stage, mt activity was significantly reduced (P<0.05). ROS levels significantly increased in vitrified embryos at morulaand blastocyst stages (P<0.001). Mt/ROS colocalizationsignificantly increased in vitrifiedmorulae. Numbers of analyzed embryos per group are indicated on the top of each histogram. Student’s t-Test: a,b P<0.05; c,d P<0.001.

Mitochondrial activity and intracellular ROS levels were evaluated at the equatorial plane of embryos which were vitrified at the morula or blastocyst stage, having a round shape and thus allowing the confocal quantification software set-up in areas describing continuous surfaces as for oocyte analysis [21]. Mitochondrial activity did not change upon vitrification in embryos at the morula stage, whereas it was reduced in embryos at the blastocyst stage (Figure 3, Panel a; P < 0.05). Intracellular ROS levels significantly increased in both morula and blastocyst stage embryos (Figure 3, Panel b; P < 0.001). Mitochondria/ROS colocalization significantly increased (P < 0.05) in vitrified versus control embryos in embryos at the morula stage whereas it did not change in embryos at the blastocyst stage (Figure 3, Panel c). In Figure 3, representative samples of mt/ROS colocalization scatterplots for a fresh (Panel d) and a vitrified (Panel e) blastocyst, are also shown.

### Vitrification does not affect mouse embryo developmental ability

The results of in vitro embryo culture obtained in the different treatment groups (vitrified, non-vitrified but exposed and non-vitrified/non-exposed to cryoprotectants control embryos) are presented in Table 1. A total of 229 cleavage stage embryos were vitrified/warmed, and out of them 11 were lost (11/229; 4.8%). From the remaining 218 embryos, 202 survived vitrification (202/218; 92.7%) and 180 developed further to expanded blastocysts during in vitro culture (180/202; 89.1%). In the group of embryos non-vitrified but exposed, 86.6% of the embryos developed to expanded blastocysts (65/75). In the group of control embryos (non-vitrified/non-exposed), 91.4% of the embryos developed to expanded blastocysts (75/82).

**Table 1:** In vitro developmental ability of vitrified mouse embryo

**Groups**	**N° (%) of cleavage stage embryos**	**N° (%) of embryos developed in vitro to expanded blastocysts**
*Vitrified embryos*	202	180 (81.9)
*Non-vitrified embryos exposed to cryoprotectants*	75	65 (86.6)
*Non-vitrified/non-exposed embryos (controls)*	82	75 (91.4)

Chi-square test: NS.

## Discussion

Vitrification slightly affected embryo morphology by increasing the percentage of embryos showing low grade (less than 20%) blastomere cytofragmentation. Embryo fragmentation is clinically considered as an indicator of reduced embryo viability and developmental ability, reduced number of high quality embryos available for establishing pregnancies, thus as a relevant problem for assisted reproduction outcome. In fresh embryos, blastomere fragmentation has been reported to be associated to apoptosis [24,35,36] or to apoptosis and necrosis [37]. A recent study [36] revealed novel early transcription mechanisms by which maternal genotype affects cytofragmentation by alterating regular cytoskeletal functions. In the present study, in which embryos were examined no more than two hours after warming (including staining procedures), these mechanisms could be only hypothesized. Thus, it can be concluded that vitrification increases only mild cytofragmentation, thus allowing the preservation of embryo blastomeres integrity, and not reducing the number of embryos available for transfer.

Vitrification increased low level chromatin damage P < 0.05). Moreover, only intermediate stages of development were affected. Although not many studies have been published so far, our observations are in line with results of previous studies demonstrating, with different methods, lower damaging effects of vitrification compared with slow freezing on embryo chromatin integrity and function. Vutyavanich et al., 2009 [38] reported significantly higher average number of nuclei in blastocysts derived from embryos vitrified at the 2-cell stage, and cultured in vitro, compared with those obtained after slow freezing. Other studies examined the effects of cryopreservation methods on DNA integrity and stability, as assessed by TUNEL test. Tsang and Chow, 2010 [39] reported significant reduction of DNA integrity after both procedures. AbdelHafez et al., 2011 [40], reported higher DNA damage extent in vitrified blastocyst versus in early cleavage stage vitrified embryos, may be attributable to differences in blastocoel shrinkage after exposure to vitrification solutions. To our knowledge, this is the first study including all developmental stages and comparing stage-specific effects of vitrification on chromatin integrity of mouse embryos.

Bioenergy/oxidative stress analysis can be performed with several molecular and biochemical methods [41]. Global assessment strategies, such as OMICS technologies, such as Transcriptomics, Proteomics, Metabolomics, are becoming increasingly valuable in this area of investigation [42]. For the specific purpose of assisted reproduction, particularly for research on oocytes and embryos, confocal imaging allows global qualitative and quantitative evaluation of bioenergy/redox parameters in individual samples, also enabling the localization and quantification of functional aberration. As for parameters of cytoplasmic maturity, reduced percentages of embryos showing mt P/P distribution pattern were observed only at early stages of development (4/16-cell stage, P < 0.001) compared with fresh control embryos. Moreover, at any stage of development the rate of vitrified embryos showing P/P pattern never dropped below 50% and in total samples, more than 70% of embryos retained this mt pattern. The qualitative analysis of fluorescence, indicative of mt activity, conducted on fresh 4/16-cell stage embryos demonstrated that: 1) mt localization was perinuclear and pericortical, 2) there were blastomeres with intense mt activity while others with almost no activity, and 3) spots of intense mt activity were evident at the level of blastomere cell junctions. This observation is in agreement with those reported by Van Blerkom, 2009 [43], who showed that mt activity can influence or can be influenced by intercellular contacts. Our observations are in agreement with a previous study by Zhao et al., 2009 [8] who reported that in fresh 2PN mouse embryos stained with JC-1, red colored mitochondria (high Δψ) were distributed primarily around pronuclei and along the cell membrane whereas in vitrified-warmed 2 PN embryos, red mt were greatly diminished with green mt (low Δψ) evenly distributed throughout the cytoplasm. At the same time, these authors found that the proportion of fresh 2PN embryos with normal aggregation of high Δψ mt (84%) was significantly higher than that of vitrified 2 PN embryos (27%). Observed altered mt distribution could be due to modifications of cytoskeletal elements which have been reported to be involved in cellular movement on a rapid timescale of these organelles [18], other cytoplasmic components, such as endoplasmic reticulum [18], or to modifications of specific proteins involved in mt anchoring to cytoskeletal microfilaments or microtubules [44]. Previous studies in the mouse reported no significant differences after vitrification in microfilament distribution in zygotes, 2 cell embryos, morulae and blastocysts [39] and in microtubule formation in 2PN embryos [8].

In embryos at the morula and blastocyst stage, the qualitative analysis showed that mt compartimentalization, which at these stages is indicative of developmental stage-dependent acquisition of blastomeres cytoplasmic maturity, was not affected by vitrification. This observation could be related to major cryotolerance but also to a greater difficulty in visualizing mt distribution modification in embryos at these stages due to reduced blastomere cytoplasmic size. Qualitative analysis also showed that mt activity of trophoectodermal cells in blastocyst stage embryos is more intense than that observed in the cells of the inner cell mass and that blastomeres showing strong mt activity were located nearby the blastocoelic cavity. This observation is in agreement with those reported by Van Blerkom, 2011, [7] who showed that the maintenance of the blastocoele and its rapid recovery after collapse and hatching phase, are morphodynamics activities that require huge production of ATP from the cells of the trophoectoderma. Instead, cells in the inner cell mass, which are not involved in these activities, appear to be metabolically quiescent in these developmental phases. Quantitative analysis of ATP production in mouse blastocysts showed that approximately 80% of ATP produced by the embryo is from the trophoectoderma and that the number of mt observed by confocal laser microscopy are located well below in the inner cell mass [7].

Quantification analysis in the present study was performed in embryos at the morula and blastocyst stage. A statistically significant reduction of MitoTracker fluorescence intensity was found in vitrified blastocysts compared with their fresh counterparts, indicating significant reduction of mt activity at this stage of development. In embryos at the morula stage, vitrification had no effects on mt activity. More interestingly, both in embryos at the morula and blastocyst stage, significant increase of DCF fluorescence intensity, indicative of an excess production of ROS, was found after vitrification. This finding could be interpreted considering that the conditions used for the vitrification method could have resulted in the onset of oxidative stress condition. This observation could be consistent with the reported up-regulation of genes involved in the mechanisms of oxidative stress (Hsp70, MnSOD, CuSOD) in mouse vitrified embryos [39]. Increased oxidative stress in vitrified/warmed embryos in the present study, kept in vitro no more than two hours after warming, could be an initial effect, as suggested by Tsang and Chow, (2010; [39]) who reported that stress-related gene expression dropped down to normal levels within 7 hours after warming. Colocalization of intracellular free radicals (ROS) and actively respiring mt has been reported as indicative of higher ATP turnover resulting from a more intense mt activity and thus indicative of healthy cell conditions in ovine in vivo matured ovulated metaphase II oocytes [21] and in hepatocytes. By this analysis, it came out that mt/ROS colocalization was not affected by vitrification in blastocyst stage embryos and it was increased in embryos at the morula stage, may be due to increased ROS generation. To our knowledge, this is the first study reporting mt/ROS colocalization, objectively expressed as Pearson’s correlation coefficient, for the comparison between fresh and vitrified mouse embryos.

Taken together our data allow to confirm that morula and blastocyst are good stages from the standpoint of embryo viability after vitrification. In vitrified morulae, only low level chromatin damage was found and bioenergy/redox parameters were positively affected. Infact, mt pattern was not affected and increased oxidative activity and consequent increased mt/ROS colocalization were found. In vitrified blastocysts, neither nuclear chromatin nor mt pattern were affected; a significant reduction of mt activity was found but ADU absolute values remained at consistent levels (395.1 ± 89.3 versus 522.4 ± 176.8 for vitrified vs fresh, respectively), indicating that vitrified embryos retained/kept a good/substantial part of mt activity. As well, in vitrified blastocysts increased respiratory/oxidative activity was found, as observed by increased ROS generation.

## Conclusions

The vitrification technology only slightly affects embryo morphology, chromatin integrity and energy/oxidative status in a developmentally-related manner. This embryo cryopreservation methods is of great scientific and clinical interest and application in both human and animal assisted reproduction. Global assessment strategies for embryo quality evaluation, such as confocal 3D imaging, can significantly contribute to the improvement of routine use crypreservation protocols, as well as to identify appropriate conditions to preserve nuclear and cytoplasmic integrity and competence to ensure proper embryonic development in the uterine environment and that the pregnancy could come to term. This study shows for the first time the joint assessment of mitochondrial activity and levels of ROS in mouse embryos in relation to their developmental stage and the application of a vitrification procedure.

## Competing interests

The authors declare that they have no competing interests.

## Authors’ contributions

Conceived and designed the experiments: SC, MED, NAM, BS, GML. Performed the experiments: NAM, BS, SC, MED. Analyzed the data: NAM, SC, MED, BS, RAC. Contributed reagents/materials/analysis tools: SC, MED, GML. Wrote the paper: NAM, SC, MED, BS, RAC, GML. Experiments supervision and critical reading of the manuscript: MED, NAM, RAC, SC. All authors read and approved the final manuscript.

## Supplementary Material

Additional file 1: Figure S1 Percentages of embryos showing either 0 to 20% (Panel A: grade B) or >20% (Panel B: grade C) chromatin damage. Numbers of analyzed embryos per group are indicated on the top of each histogram. Chi square test: within each stage: a,b P < 0.001; c,d P < 0.05; between stages: (*) P < 0.05. Click here for file
