# Supplementary figures and images for: Vitrification preserves chromatin integrity, bioenergy potential and oxidative parameters in mouse embryos
Source: Reprod Biol Endocrinol. 2013 Apr 3;11:27. doi: 10.1186/1477-7827-11-27 (PMC3652727; doi:10.1186/1477-7827-11-27)

A

**Embryos showing 0-20 % (grade B)  
chromatin damage (%)**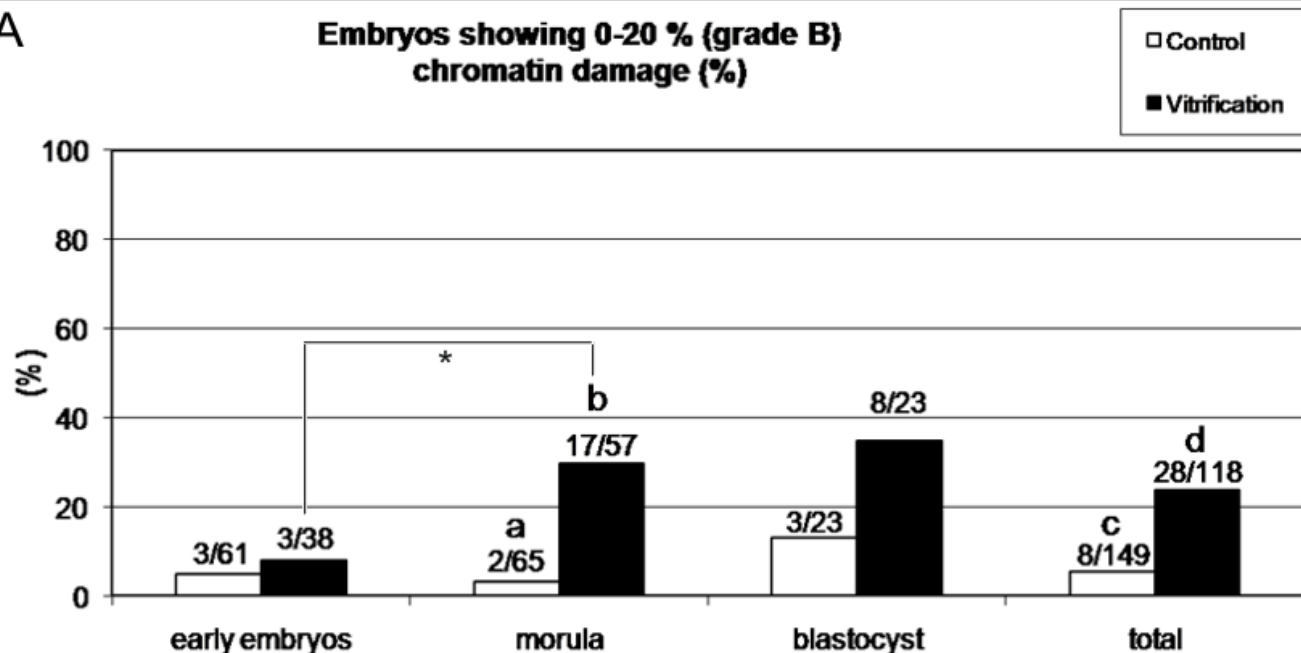

B

**Embryos showing > 20 % (grade C)  
chromatin damage (%)**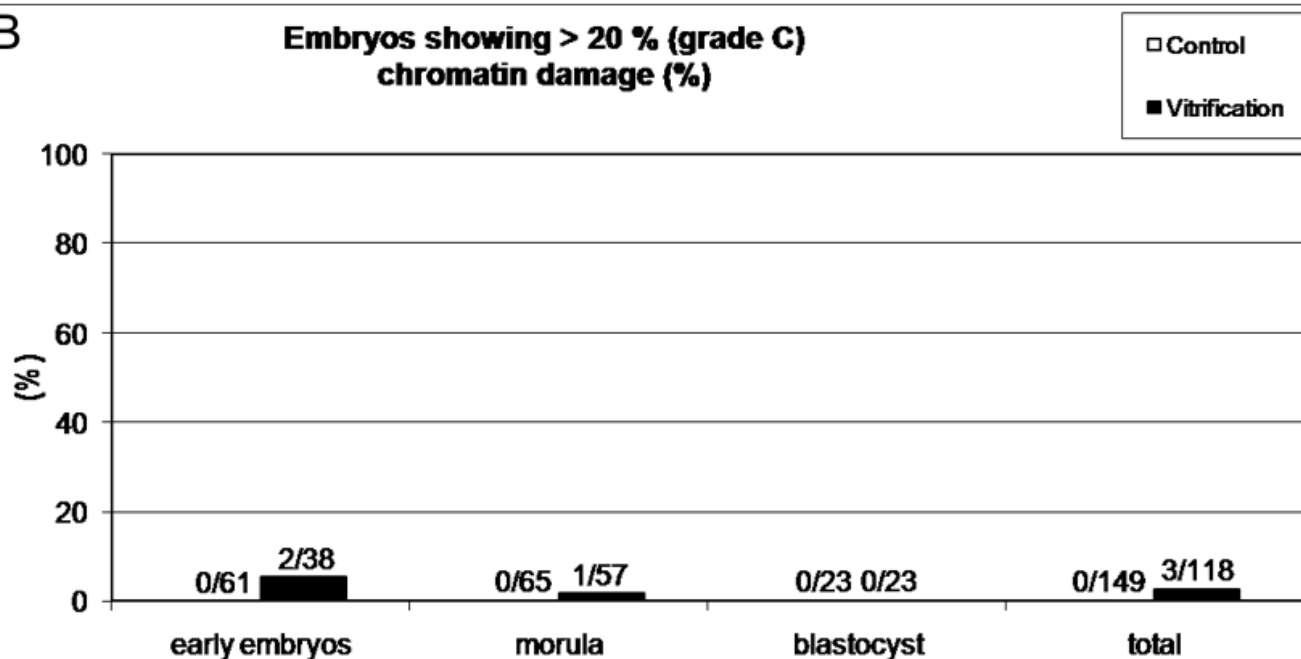

Supplement: Additional file 1: Figure S1 — Percentages of embryos showing either 0 to 20% (Panel A: grade B) or >20% (Panel B: grade C) chromatin damage. Numbers of analyzed embryos per group are indicated on the top of each histogram. Chi square test: within each stage: a,b P < 0.001; c,d P < 0.05; between stages: (*) P < 0.05. [file 1477-7827-11-27-S1.pdf]
